# Congenital Blood Cyst of a Child: A case report and review of literature

**DOI:** 10.18295/squmj.1.2024.002

**Published:** 2024-05-27

**Authors:** Rachid Kaddoura, Issam El Rassi, Zainab Al Awadi, Mohamed Kasem

**Affiliations:** 1 College of Medicine, Mohammed Bin Rashid University of Medicine and Health Sciences, Dubai, United Arab Emirates; 2 Al Jalila Children’s Speciality Hospital, Dubai, United Arab Emirates

**Keywords:** Cyst Fluid, Pulmonary Valve, Pulmonary Artery, Paediatrics, Cardiac Tumors, Cardiology

## Abstract

Blood-filled cysts of the heart valves are frequently reported at postpartum autopsies of infants. They are seen as round nodules mostly in the paediatric age group in infants less than 2 months of age and disappear spontaneously within 6 months of life. We report a unique case of an 11-month-old girl who presented at a tertiary healthcare hospital in 2022 with a blood-filled cyst on the posterior leaflet of the pulmonary valve that was successfully treated. This case report highlights the characteristics and course of a paediatric patient with blood-filled cysts. Further studies are yet needed to better understand the diagnostic approaches to blood-filled cysts as well as treatment modalities to fill the gap in clinical settings.

Blood-filled cysts (BFCs) are rare benign tumours mainly reported as cardiac tumours.^1^ Since primary cardiac valve tumours are very uncommon, autopsy studies have provided much of the current detailed information available in the literature.^2^^,^^3^ Numerous parameters—such as tumour classification, location size, growth rate and susceptibility to embolise—affect the overall clinical presentation. Both myxomas and papillary fibroelastomas are the pathological conditions most likely to be related to embolism, with the latter making up most primary valve tumour types.^3^^,^^4^ Moreover, lipomas, myxomas and rhabdomyomas are all reported as primary cardiac tumours as well.

BFCs of the heart valves were first reported in 1844 by Elsässer.^5^ BFCs are often found on the atrioventricular valves of newborn infants’ necropsies. They are usually seen as small, rounded, multiple nodules on the atrial surfaces of the atrioventricular valves, but are also seen less often on the ventricular surfaces of the semilunar valves.^1^–^3^ In this report, we present a rare case of a paediatric patient with a BFC on the pulmonary valve leaflet.

## Case Report

An 11-month-old girl presented to her general paediatrician (GP) at a tertiary healthcare hospital for a non-cardiac cause at the beginning of 2022. She was noted to have a loud systolic heart murmur in the pulmonary area. The child was otherwise well, had no previous history of infections and her clinical examination findings were normal.

The echocardiogram demonstrated severe right ventricular outflow obstruction due to a possible cyst on the posterior leaflet of the pulmonary valve. The valve itself looked normal and there was post-stenotic dilatation of the main pulmonary artery. The gradient across the pulmonary valve had a peak of 65 mmHg.

**Figure 1:**
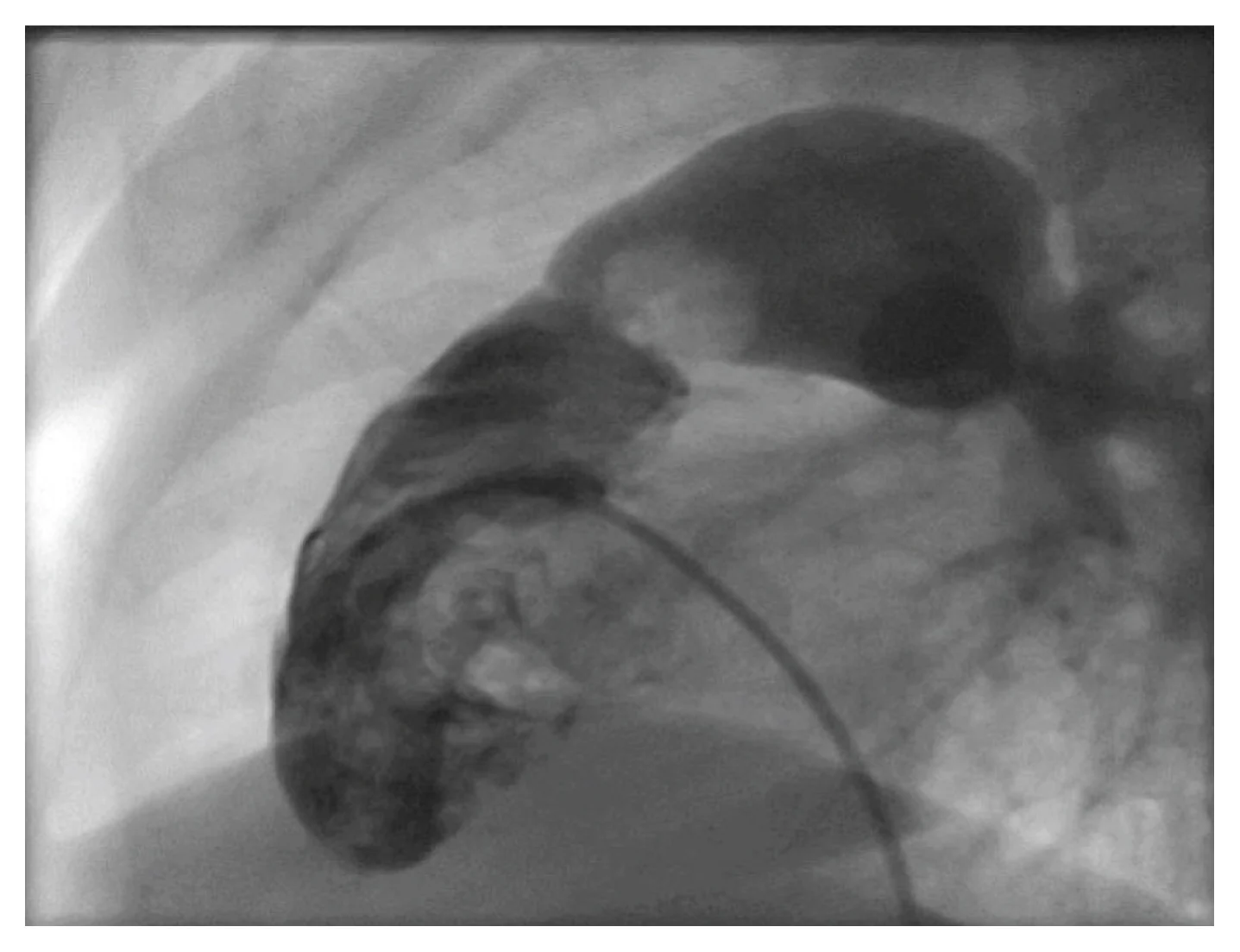
Right heart catheter in an 11-month-old girl showing circumscribed rounded cysts.

The patient underwent a right heart catheterisation. The angiogram showed a cyst fixed on the surface of one of the pulmonary valve leaflets [Figure 1]. The cyst was mobile with the leaflet, but not causing any regurgitation. No ballooning was done and the patient had a chest computed tomography (CT) scan which showed a filling defect in the main pulmonary artery. The CT scan showed normal distal pulmonary arteries and branches, as well as normal lung parenchyma, mediastinum and no lymphadenopathy. A comprehensive infectious and immunological assessment showed no underlying disease.

**Figure 2:**
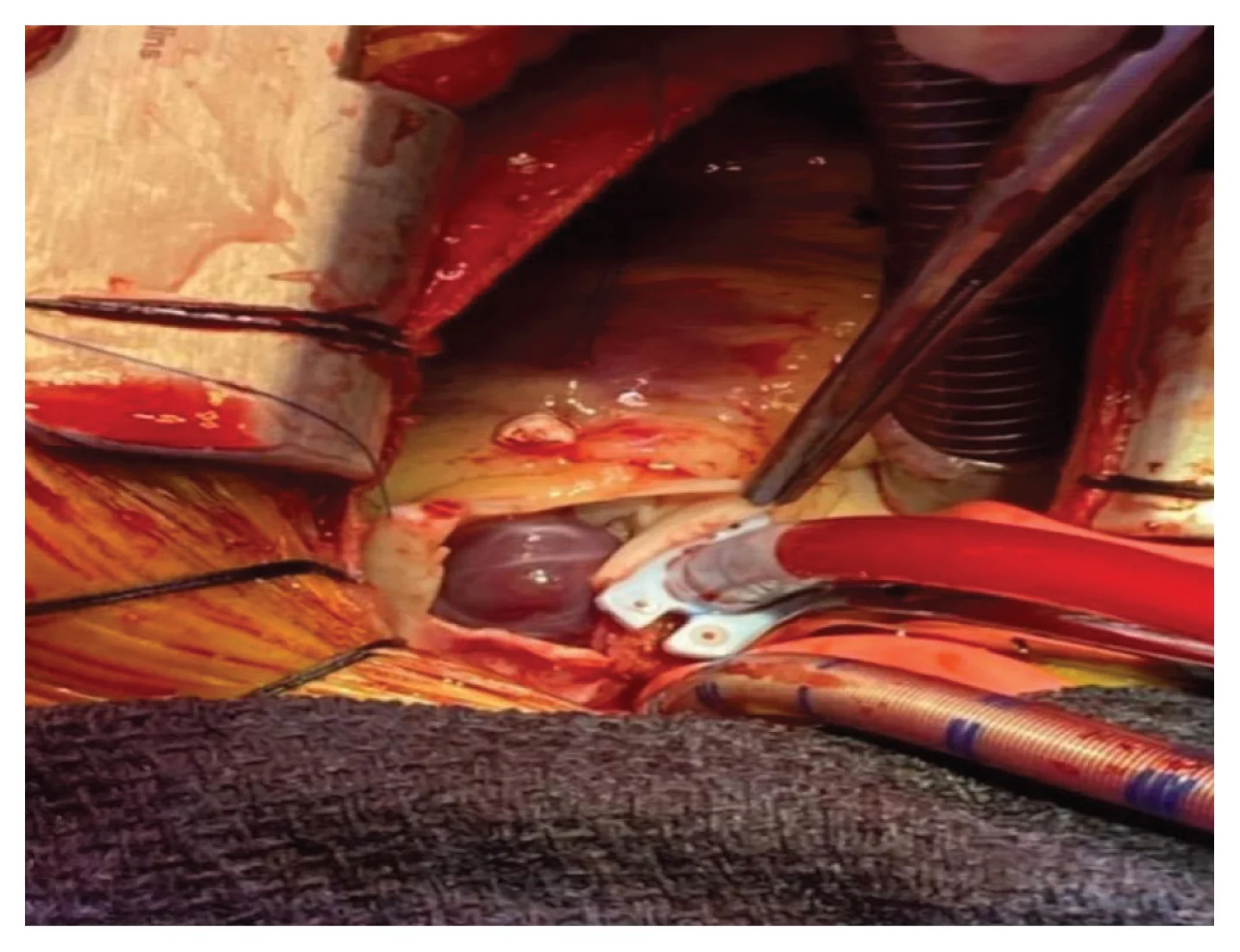
Surgical removal of a blood-filled cyst from an 11-month-old girl.

After discussions, it was decided to surgically remove the cyst [Figure 2]. The surgical procedure was uneventful. Resection of the whole cyst that was attached to the posterior leaflet of the pulmonary valve was performed, and the gradient decreased to less than 15 mmHg, with moderate pulmonary valve regurgitation.

**Figure 3:**
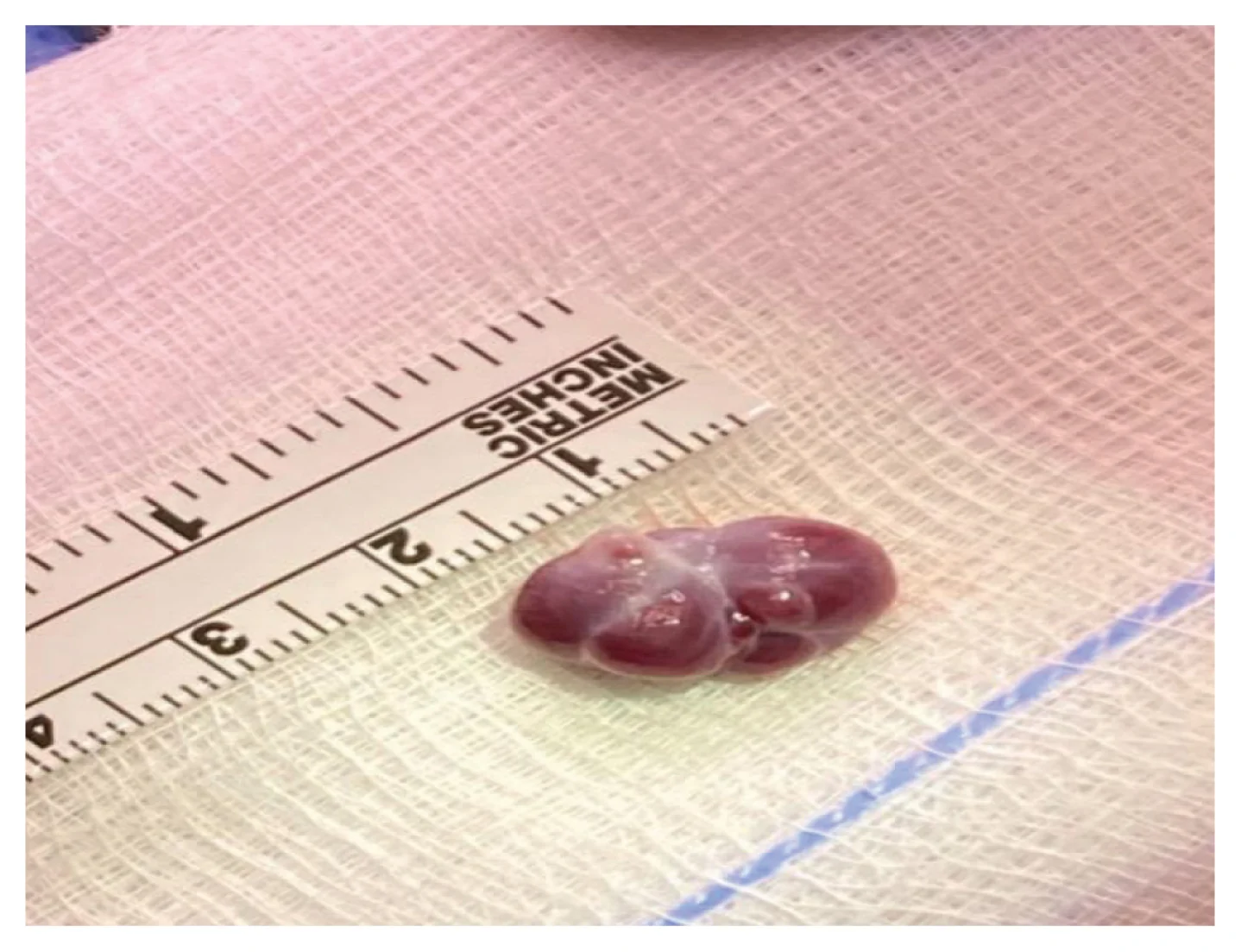
Gross appearance of the blood-filled cysts that was surgically removed from an 11-month-old girl.

The histopathology of the cyst showed a 1 × 0.5 × 0.3 cm multiloculated cyst, filled with blood and composed of a thin fibrous wall with focal myxoid changes [Figure 3]. The child made full recovery. At the age of three, only a 12-mmHg peak gradient at the pulmonary valve was observed, with moderate regurgitation and no new cysts were noted on any of the heart valves.

Informed and written consent for the patient’s procedure and publication purposes for this case report was obtained from the parents.

## Discussion

This report presents a unique case of a paediatric patient with a BFC on the posterior leaflet of the pulmonary valve that was successfully managed and treated. Unlike the paediatric age group, singular valvular BFCs are rarely reported in older children and adults, this is attributed to the fact that the cysts spontaneously regress in most patients as they age.^6^ Several previous reports described 12 BFCs of the pulmonary valve that were treated successfully by surgical resection.^5^–^12^

BFCs present with symptoms of severe valvular stenosis due to the outflow obstruction, as well as regurgitation presenting with signs of cyanosis, although they have mostly been reported to be asymptomatic and only discovered incidentally usually on CT scans done for non-cardiac reasons, especially in the infant age group.^6^^,^^12^ The patient presented in this case report, initially went to the GP with no cardiac symptoms, and the leading cause of the BFCs discovery was due to a loud systolic murmur detected by the GP. Following that, she was referred to the cardiology department and an echocardiogram was done which identified the BFC. Echocardiogram is considered to be the gold standard for the diagnosis of BFCs, and in rare cases where a thrombus or bacterial vegetations are suspected, contrast echocardiogram might help differentiate it from cardiac cysts.^13^

To date, it is still unknown where BFCs in cardiac valves arise from. Several animal studies revealed that 20% of all animal hearts contained BFCs, highlighting its high prevalence.^14^ Regarding the development of BFCs, adult cases have been attributed to blunt trauma to the chest and complications during valvular surgeries; however, their cause in the paediatric age group is still unknown.^13^ Tsutsui *et al*. suggested that BFCs may originate during the development of valves in early embryogenesis or during the early period of life from blood entrapped in valvular crevices or tiny invaginations during development; therefore, a neonate with normal echocardiogram findings can still develop BFCs in early infancy or childhood.^15^ Although, this process is very unlikely to occur at later stages during adulthood. Another hypothesis suggests that BFCs are primarily due to haematoma formation as a result of blocked small vessels.^12^ Furthermore, based on the findings of the histological and ultrastructural analysis, BFCs could be due to the expansion of thin-walled valvular arteries in response to mechanical stress caused by the pressure gradient when atrioventricular valves are closed, developing a cyst.^14^ However, the presence of BFCs in low-pressure structures like the pulmonary valve cannot be explained by the mentioned theory. Therefore, the mentioned hypotheses are quite hard to confirm and the definite formation of a BFC is not yet well established in the current literature.

In terms of management, Paşaoğlu *et al*. suggested surgically removing the BFCs in the heart at the time of diagnosis independent of the patient’s symptoms.^10^ This was the course of action for the current patient. On the other hand, Dencker *et al*. encouraged a more conservative approach in asymptomatic patients and stated that surgical approaches should be kept for symptomatic patients or if the cyst leads to cardiac dysfunction.^13^ Surgical interventions are usually done in order to rule out malignancy and risk of strokes. Pharmacological therapies including anticoagulants and beta-blocker use are still controversial with very little evidence available in the current literature.^13^ This emphasises the need for more research exploring the outcomes of different management approaches.

## Conclusion

Although several theories have been postulated regarding the origin of BFCs, it remains unknown. BFCs are rare and are seen as small round nodules on imaging modalities. However, a better understanding of the diagnostic approaches to BFCs as well as treatment modalities is required to ensure an overall better prognosis in both the adult and paediatric age groups.

## References

[1] Yada M, Nishina T, Sato S, Ueda Y, Yamanaka K (2021). Cardiac blood cyst with concomitant papillary fibroelastoma. J Cardiol Cases.

[2] Wang Y, Wang X, Xiao Y (2016). Surgical treatment of primary cardiac valve tumor: early and late results in eight patients. J Cardiothorac Surg.

[3] Edwards FH, Hale D, Cohen A, Thompson L, Pezzella AT, Virmani R (1991). Primary cardiac valve tumors. Ann Thorac Surg.

[4] Gopaldas RR, Atluri PV, Blaustein AS, Bakaeen FG, Huh J, Chu D (2009). Papillary fibroelastoma of the aortic valve: operative approaches upon incidental discovery. Tex Heart Inst J.

[5] Elsässer C (1844). Bericht über die ereignisse in der gebäranstalt des Catherinen-Hospital in Jahre 1844. Med Correspondenzblatt.

[6] Gallucci V, Stritoni P, Fasoli G, Thiene G (1976). Giant blood cyst of tricuspid valve. Successful excision in an infant. Br Heart J.

[7] Liese GJ, Brainard SC, Goto U (1963). Giant blood cyst of the pulmonary valve. Report of a case. N Engl J Med.

[8] Cumming GR, Ferguson CC (1965). An elusive tumor of the pulmonary valve associated with a coronary arteriovenous fistula. J Thorac Cardiovasc Surg.

[9] Sakakibara S, Katsuhara K, Iida Y, Nishida H (1967). Pulmonary subvalvular tumor. Dis Chest.

[10] Paşaoğlu I, Doğan R, Demircin M, Bozer AY (1993). Blood cyst of the pulmonary valve causing pulmonic valve stenosis. Am J Cardiol.

[11] Minato H, Manabe T, Masaki H, Kawahara Y (1997). Blood cyst of the pulmonary valve in an adult: report of a case and review of the literature. Hum Pathol.

[12] Park MH, Jung SY, Youn HJ, Jin JY, Lee JH, Jung HO (2012). Blood cyst of subvalvular apparatus of the mitral valve in an adult. J Cardiovasc Ultrasound.

[13] Dencker M, Jexmark T, Hansen F, Tydén P, Roijer A, Lührs C (2009). Bileaflet Blood Cysts on the Mitral Valve in an Adult. J Am Soc Echocardiogr.

[14] Marcato PS, Benazzi C, Bettini G, Masi M, Della Salda L, Sarli G (1996). Blood and serous cysts in the atrioventricular valves of the bovine heart. Vet Pathol.

[15] Tsutsui JM, Pommerantzeff P, Pinto IM, Rochitte CE, Mathias W (2008). Characterization of blood-filled cyst by contrast echocardiography and computed tomography. J Am Soc Echocardiogr.

